# Mucoepidermoid Carcinoma of the Hard Palate: A Case Illustrating Grading Uncertainty and Bone-Sparing Surgical Management

**DOI:** 10.7759/cureus.115250

**Published:** 2026-08-26

**Authors:** Maryam Ellam, James Barraclough, Amal Suresh, Danny Adam, Neil Sahasrabudhe

**Affiliations:** 1 Surgery, Lancaster University, Lancaster, GBR; 2 Maxillofacial Surgery, Royal Blackburn Teaching Hospital, Blackburn, GBR; 3 Oral and Maxillofacial Surgery, Royal Blackburn Teaching Hospital, Blackburn, GBR; 4 Pathology, Royal Blackburn Teaching Hospital, Blackburn, GBR

**Keywords:** bone-sparing surgery, hard palate, histological grading, maml2, minor salivary gland tumour, mucoepidermoid carcinoma

## Abstract

Mucoepidermoid carcinoma (MEC) of the hard palate presents diagnostic and surgical challenges, particularly regarding histological grading and preservation of the underlying palatal bone. Histological grading remains controversial because of multiple grading systems and inter-observer variability.

A 42-year-old man presented with a painless 10-12 mm right paramedian hard-palate swelling that had been present for approximately 12 months, with slight enlargement over the preceding two months, identified incidentally during routine dental examination. Biopsy confirmed MEC. MRI and CT demonstrated a 15 × 12 × 5 mm lesion with cortical thinning but no cortical breach or definitive bone invasion. Following multidisciplinary team discussion, wide local excision with periosteal elevation and preservation of palatal bone was performed because of the small, well-circumscribed lesion, low-risk histological features, absence of radiological or intra-operative bone invasion, and importance of preserving palatal function. Final histology demonstrated low-grade features by AFIP criteria and intermediate-grade features by the Brandwein system, with no bone invasion.

This case illustrates the challenges of grading discordance and integrating histopathological, radiological and intra-operative findings when considering bone-sparing surgery. In selected low-risk palatal MECs without evidence of osseous invasion, conservative surgery may provide good short-term functional outcomes.

Multidisciplinary interpretation of grading, imaging and intra-operative findings may support bone-sparing management in carefully selected palatal MEC.

## Introduction

Mucoepidermoid carcinoma (MEC) is the most common malignant salivary gland tumour, accounting for approximately 30-35% of cases [1]. Although the parotid gland is the most frequent site, up to a quarter of MECs arise in the minor salivary glands, with the hard palate being the most common intraoral location [1]. Palatal MECs often present as painless, slow-growing lesions and may resemble benign or inflammatory conditions, contributing to delays in diagnosis. The differential diagnosis of a persistent palatal swelling includes other minor salivary gland tumours, squamous cell carcinoma, lymphoma, benign salivary neoplasms and inflammatory or reactive lesions.

MEC exhibits a wide spectrum of biological behaviour. Low-grade tumours are typically cystic and indolent with an excellent prognosis, whereas high-grade lesions are more infiltrative and associated with regional and distant spread [1]. Accurate histological grading is therefore critical for prognostication and surgical planning. However, grading remains controversial due to interobserver variability and differences between grading systems. The AFIP system uses a points-based assessment incorporating features such as cystic component, neural invasion, necrosis, mitotic activity and anaplasia, whereas the Brandwein system incorporates additional adverse features such as lymphovascular and bone invasion and infiltrative growth. The MSKCC (Healy) system uses a predominantly qualitative assessment of architectural and cytological features, while the WHO 5th edition has adopted a simplified binary low- versus high-grade approach. These differences can result in discordant grading of the same tumour and may influence treatment decisions [2-4].

Although palatal MEC is a recognised entity, uncertainty remains regarding how histological grading, radiological assessment of bone involvement and the extent of surgical resection should be integrated in individual patients. This case is instructive because it demonstrates discordant AFIP and Brandwein grading in a small palatal MEC with radiological cortical thinning but no definitive bone invasion, in which multidisciplinary team (MDT) assessment, intraoperative confirmation of an intact periosteum and preservation of palatal bone allowed wide local excision without bony resection. The case therefore illustrates a clinically relevant decision point between oncological clearance and functional morbidity and provides an example of how multidisciplinary assessment can be used to individualise treatment in selected patients.

The optimal management of the underlying palatal bone is not established by a universal requirement for routine bone resection. Published series suggest that, in low-grade palatal MEC where pre-operative imaging does not demonstrate bone invasion, mucosal or soft-tissue excision without routine bone removal can achieve favourable local control [5]. Other studies have similarly suggested that tumour grade, stage and margin status are more important prognostic determinants than routine resection of adjacent bone [6,7]. Consequently, contemporary management may favour an individualised approach in which the decision to resect bone is based on radiological evidence of invasion, tumour biology, intra-operative findings and the anticipated functional morbidity of bony resection [8,9].

## Case presentation

History

A 42-year-old man was referred by his general dental practitioner via the two-week cancer pathway after a small, asymptomatic swelling was noted at the midline/right hard palate during a routine dental examination. The lesion was referred as a soft tissue mass inconsistent with a torus.

The patient initially attributed the lesion to irritation from spicy food consumed two weeks prior. However, at subsequent maxillofacial clinic review, he reported that a swelling had in fact been present for approximately 12 months, with slight enlargement over the preceding two months. He denied pain, bleeding, discharge, dysphagia, odynophagia, dental pain or systemic symptoms including weight loss, fever or night sweats. He was a lifelong non-smoker, did not consume alcohol and did not chew tobacco. His past medical history included asthma managed with salbutamol and steroid inhalers.

**Figure 1:**
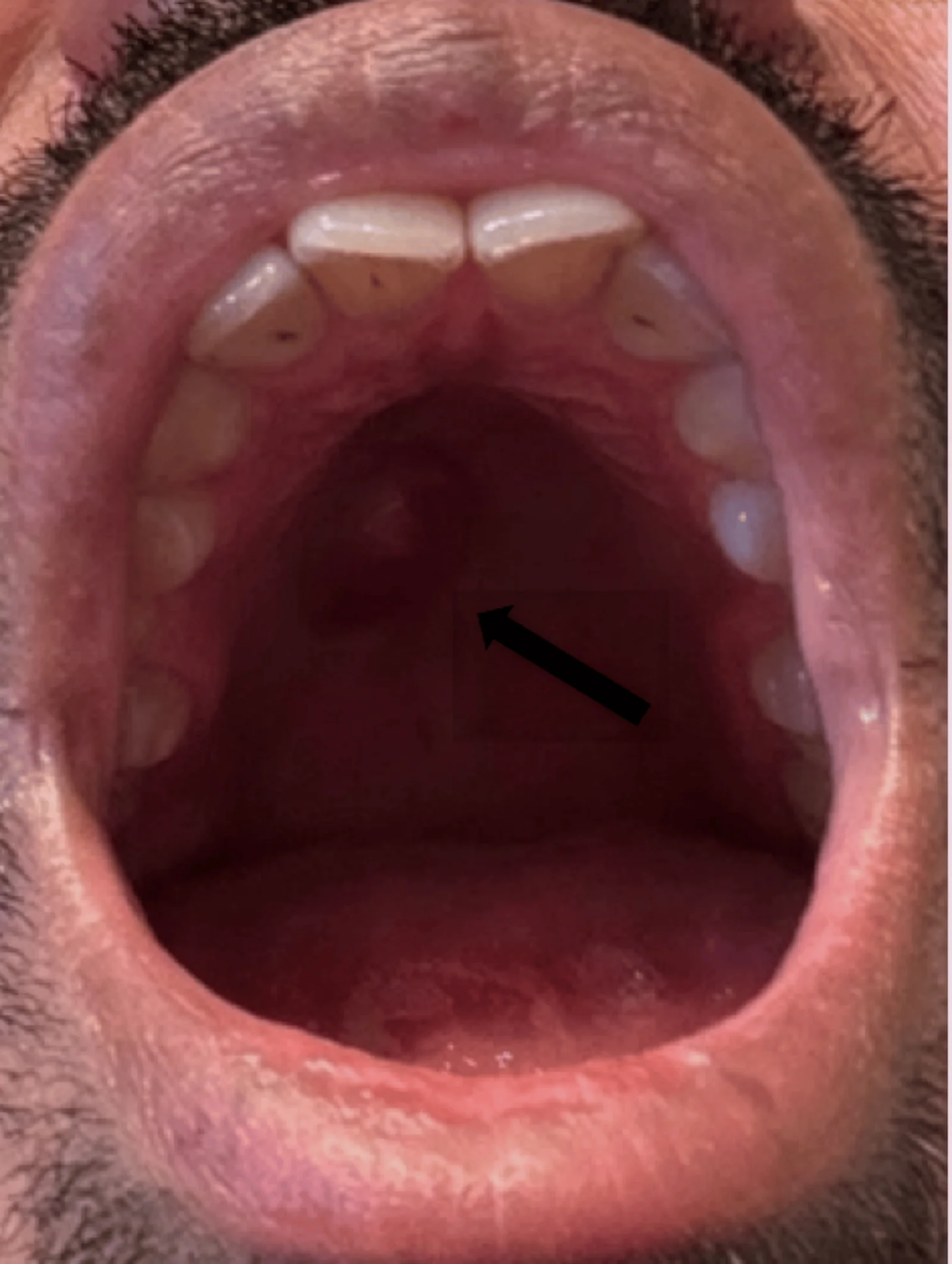
Initial clinical presentation. Clinical photograph demonstrating a right paramedian hard-palate swelling measuring approximately 10–12 mm, with an erythematous, sessile appearance and intact overlying mucosa. The arrow indicates the palatal lesion.

On examination, inspection revealed a 10-12 mm erythematous, sessile swelling on the right paramedian hard palate (Figure 1). The overlying mucosa was intact. Palpation demonstrated a cystic, fluctuant lesion with mild tenderness. There was no cervical or parotid lymphadenopathy.

Investigations

**Figure 2:**
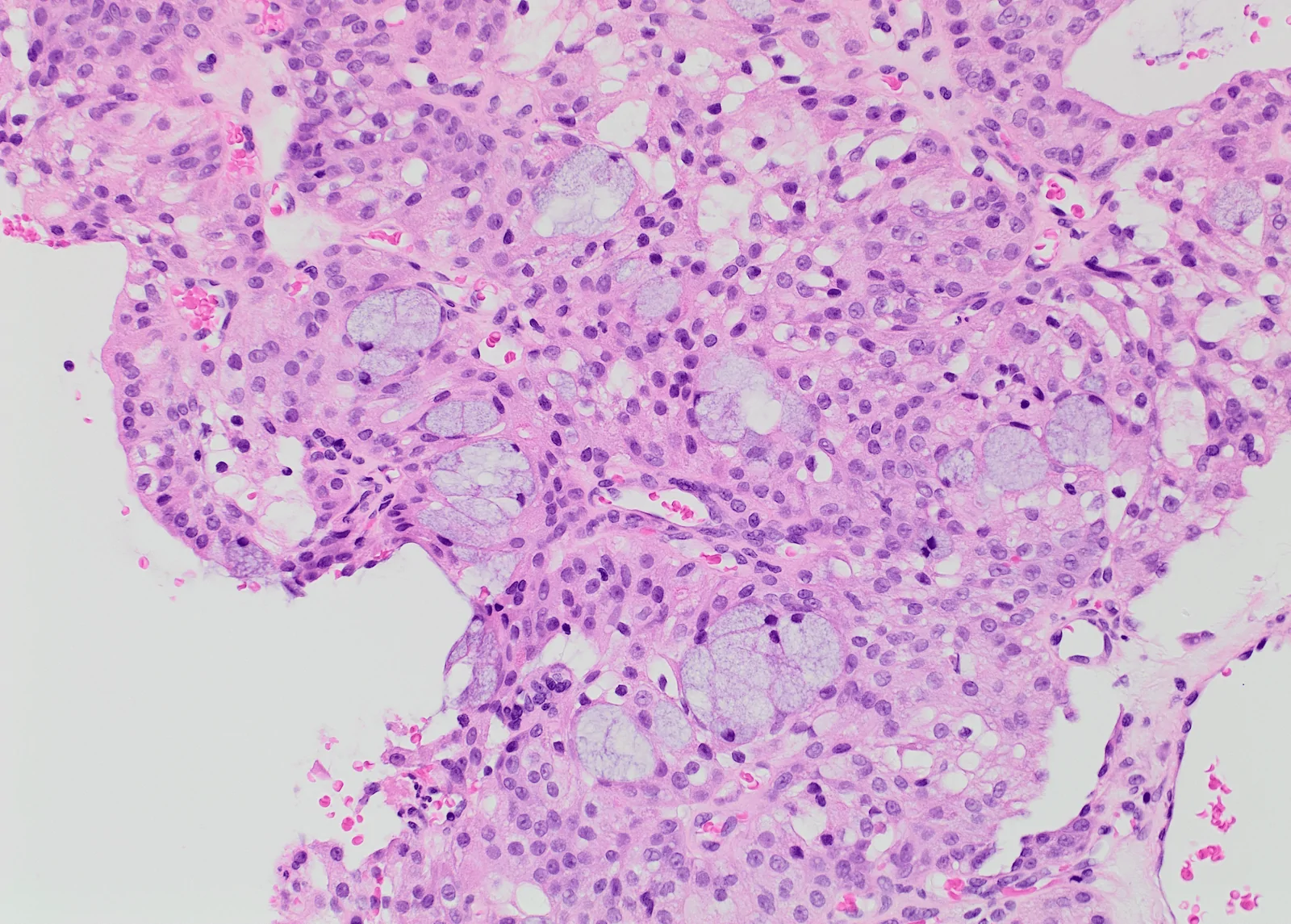
Histopathological findings. Histopathological examination demonstrating mucoepidermoid carcinoma composed predominantly of sheets of intermediate cells, with focal clear cell change and interspersed mucocytes. Haematoxylin and eosin staining, ×20 magnification.

An incisional biopsy was performed under local anaesthetic. Histopathological analysis of the incisional biopsy confirmed MEC; however, formal grading was not assigned at this stage. Definitive grading was undertaken following excision and demonstrated low-grade features by AFIP criteria and intermediate-grade features by the Brandwein system (Figure 2). 

**Figure 3:**
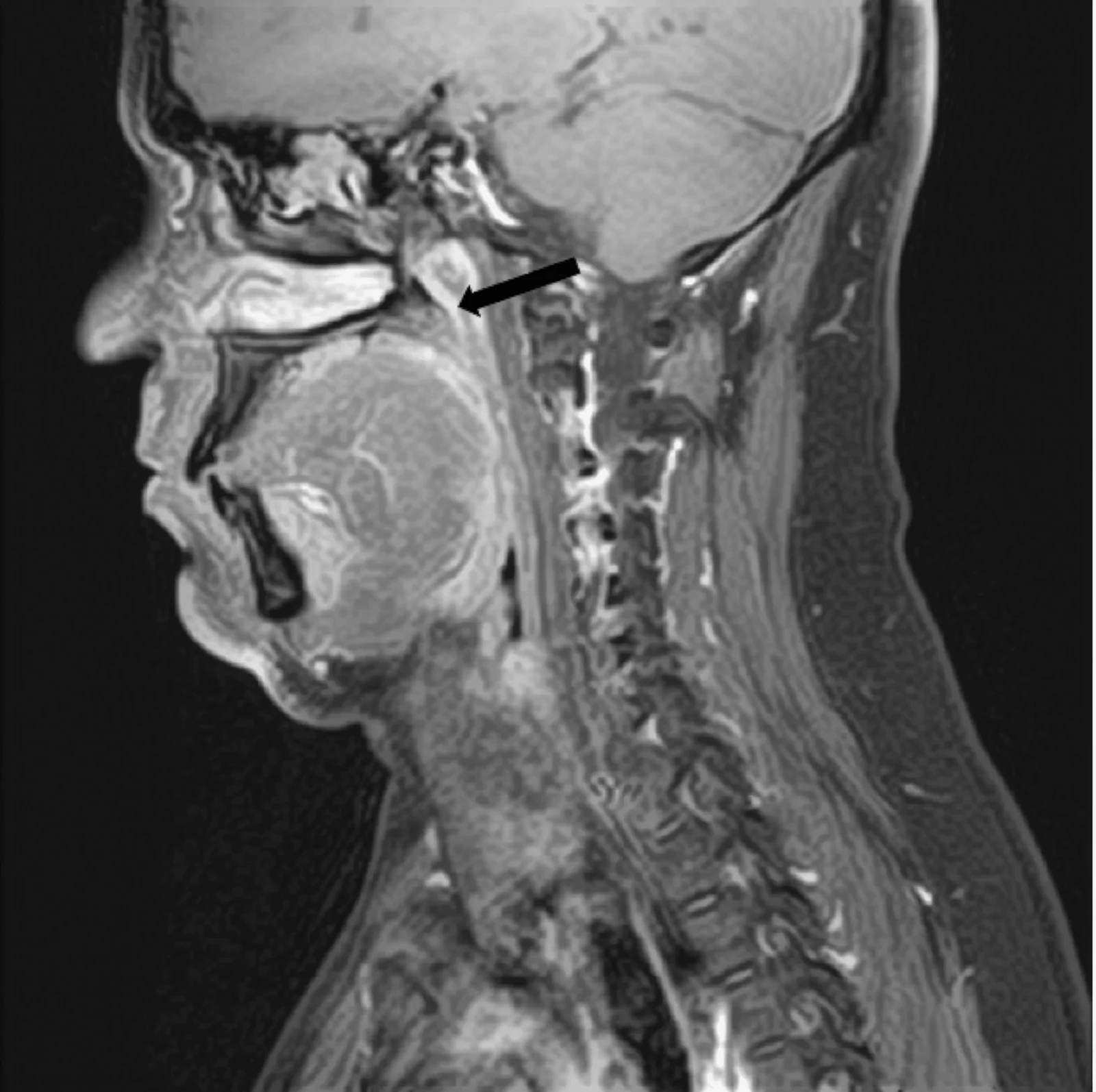
Magnetic resonance imaging findings. T1 contrast-enhanced sagittal MRI of the face demonstrating the right hard palate lesion.

Cross-sectional imaging with contrast-enhanced MRI of the face and CT of the neck and thorax demonstrated a 15 × 12 × 5 mm mucosal lesion of the right hard palate (Figure 3). 

The underlying palatal bone appeared thinned but without cortical breach or dehiscence. There was no extension into the nasal cavity, no cervical lymphadenopathy and no evidence of distant metastatic disease. These findings supported consideration of a bone-sparing surgical approach.

Management

The case was discussed at the regional Head and Neck MDT meeting. In view of the small, well-circumscribed lesion, low-risk pathological features, absence of radiological bone invasion and the importance of preserving palatal function, the MDT recommended wide local excision with an appropriate clinical mucosal margin; a specific numerical margin was not predetermined because of the lesion's small size and palatal location.

**Figure 4:**
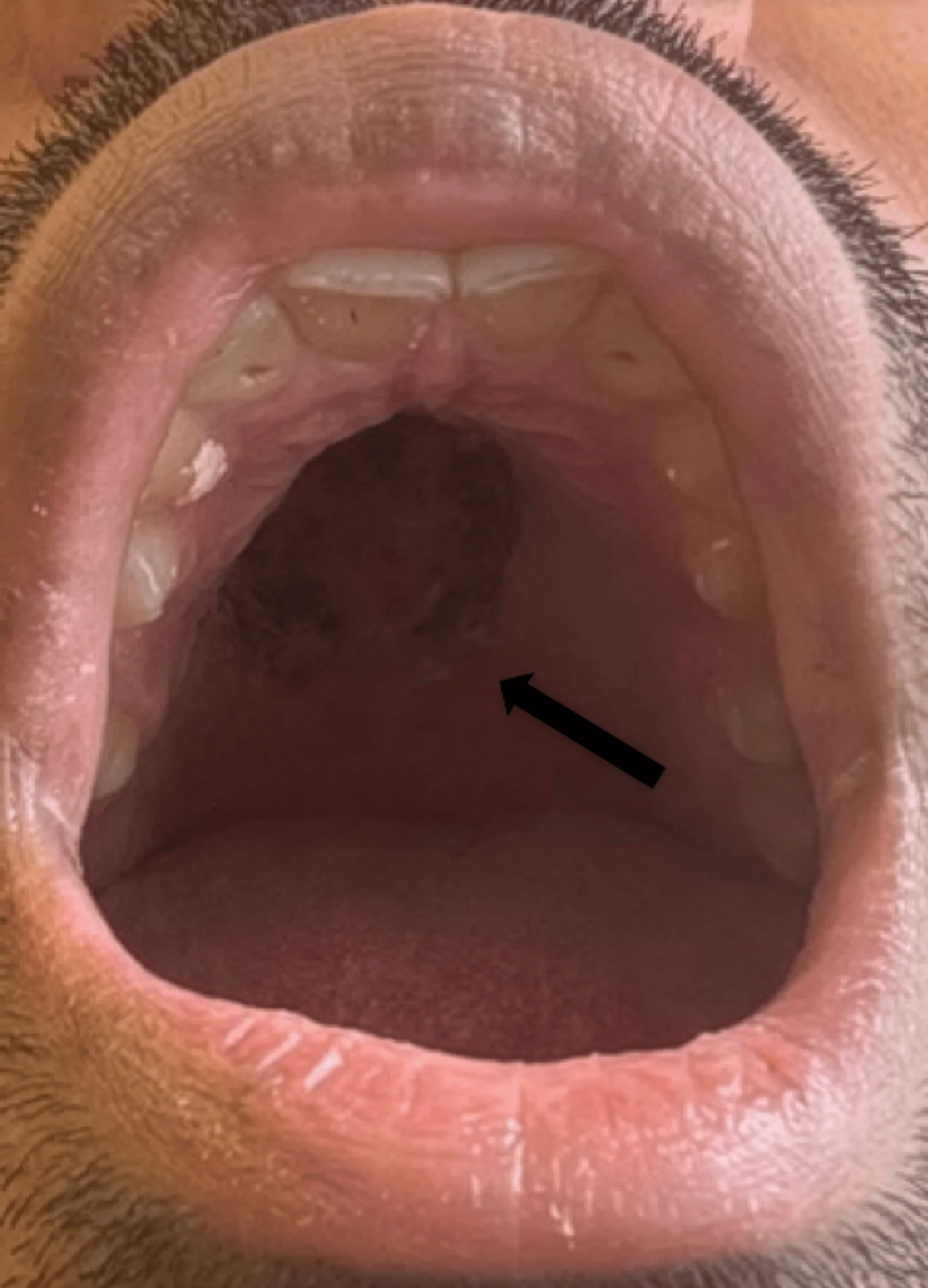
Immediate postoperative appearance. Clinical photograph demonstrating the palatal surgical site immediately following wide local excision of the lesion with preservation of the underlying palatal bone. The arrow indicates the operative site.

The patient underwent wide local excision with a planned mucosal margin and placement of a palatal cover plate secured with Coe-Pak dressing (Figure 4). Intraoperatively, the periosteum was not infiltrated and the palatal bone appeared intact and uninvolved; therefore, no bony resection was undertaken.

Following definitive excision, gross examination of the specimen demonstrated a well-circumscribed, nodular lesion. Final histology demonstrated a 6 mm MEC arising from minor salivary glands with an intracystic component of less than 20%. There was no evidence of necrosis, significant nuclear atypia, increased mitotic activity, perineural invasion or lymphovascular invasion. The tumour was graded as low-grade according to the AFIP system and intermediate-grade according to the Brandwein system. Peripheral soft tissue margins exceeded 5 mm. The deep margin was clear at 0.2 mm with a clear periosteal layer and uninvolved bony spicules present at the deep margin, suggestive of complete excision. There was no histological evidence of bone invasion.

Following discussion at the head and neck MDT, the final diagnosis was a low-grade MEC which has been completely excised, with a close deep margin for bone preservation and function. The MDT, comprising head and neck/maxillofacial surgeons, radiology, histopathology, oncology and specialist nursing representation, recommended no further treatment and clinical surveillance only. The close deep margin of 0.2 mm was considered in the context of the complete pathological and radiological assessment. Although the margin was close, it remained histologically clear, with an intact periosteal layer and no evidence of osseous invasion. Given the low-risk histological features, absence of perineural or lymphovascular invasion, lack of radiological or intraoperative bone involvement, and the morbidity associated with further palatal bone resection, the MDT considered additional surgery unlikely to provide sufficient benefit to justify the functional consequences. Clinical surveillance was therefore recommended.

Outcome

**Figure 5:**
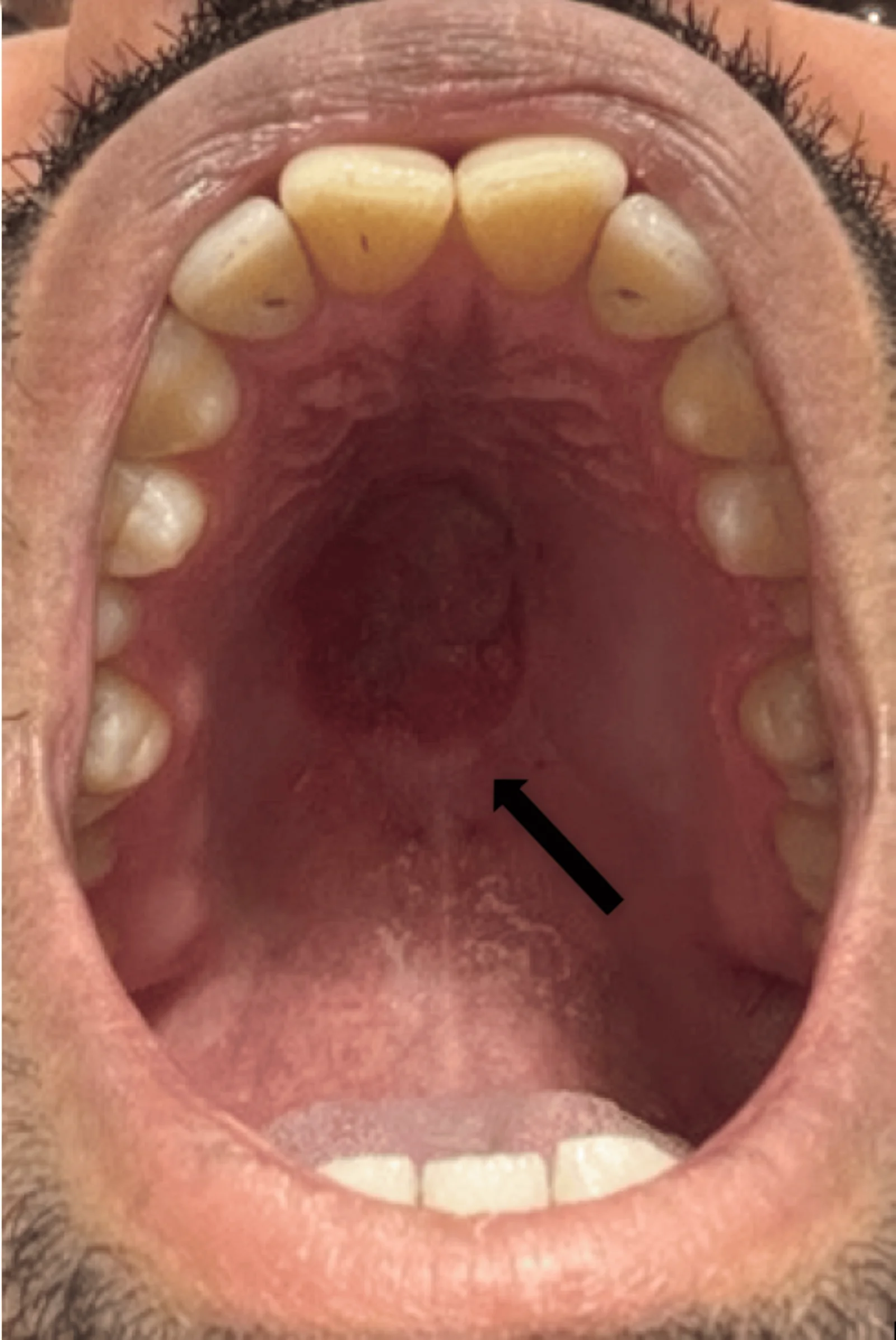
Two-month postoperative follow-up. Clinical photograph demonstrating satisfactory healing of the right paramedian hard palate two months following wide local excision, with mucosal granulation and improving oral function. The arrow indicates the healing surgical site.

**Figure 6:**
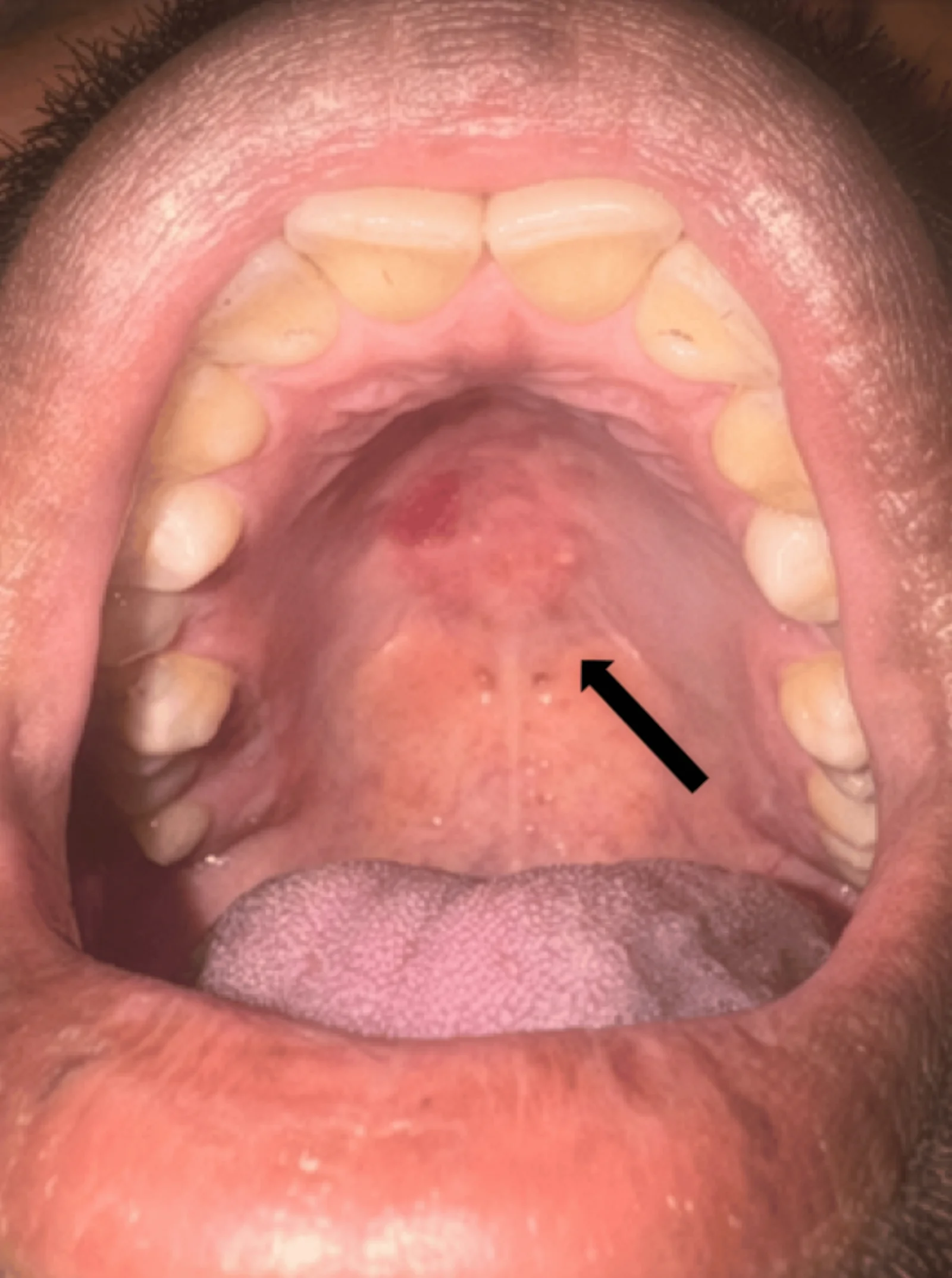
Four-month postoperative follow-up. Clinical photograph demonstrating a well-healed hard palate four months following bone-sparing wide local excision, with good mucosal remodelling and preservation of the palatal contour. The arrow indicates the previous surgical site.

Postoperative recovery was uneventful, with no postoperative bleeding, infection, wound dehiscence or oronasal fistula. Swallowing was assessed as safe prior to recommencing oral intake. Follow-up at two and four months demonstrated good healing and improving speech and oral function (Figures 5, 6), with no evidence of complication or recurrence. The patient remains under routine surveillance.

## Discussion

This case highlights three central controversies in the management of palatal MEC: uncertainty in histological grading, interpretation of radiological bone involvement, and the extent of surgical resection required. Together, these challenges emphasise the importance of integrating histopathological findings, imaging and MDT discussion when planning surgery [5,8,10]. In this patient, grading was discordant, with the tumour classified as low-grade by the AFIP system and intermediate-grade by the Brandwein system. This discrepancy is well recognised and illustrates the limitations of existing grading frameworks and the value of MDT interpretation in selecting patients suitable for bone-sparing surgery.

MEC is composed of varying proportions of mucous, intermediate and squamoid cells, resulting in a broad biological spectrum [1]. Traditional three-tier grading categorises tumours as low, intermediate or high grade, with grade strongly correlating with recurrence risk, metastatic potential and overall survival [1]. However, grading remains inconsistent, with significant inter-observer variability and limited reproducibility across systems [2].

The AFIP system is a structured, points-based model that assesses five histological parameters: proportion of cystic component, neural invasion, necrosis, mitotic activity and anaplasia [2]. It is widely used and relatively reproducible, but has been criticised for occasional under-grading, as some tumours classified as low-grade may behave aggressively.

In contrast, the Brandwein system incorporates a broader range of adverse features, including lymphovascular invasion, bone invasion, perineural invasion and infiltrative growth patterns [1]. These adverse features have been associated with higher recurrence risk and more aggressive biological behaviour in comparative grading studies [11], and as a result the Brandwein system tends to upgrade tumours compared with AFIP. This may improve sensitivity for aggressive disease but risks overestimating biological behaviour in indolent lesions. In this case, Brandwein grading classified the tumour as intermediate grade, reflecting the system’s tendency to assign higher risk categories.

The MSKCC (Healy) system is a qualitative, pattern-recognition approach based on architectural and cytological features rather than a points-based score. Comparative studies have shown variable agreement between MSKCC and other grading systems, with limited evidence of prognostic superiority over AFIP or Brandwein [3].

The WHO 5th edition classification has moved towards a binary grading model, recognising that low- and intermediate-grade MECs often have similar clinical outcomes [4]. This two-tier approach (low vs high grade) may reduce grading variability and better reflect biological behaviour, but adoption remains inconsistent in routine practice.

Grading challenges are particularly relevant in minor salivary gland tumours. Some studies suggest that AFIP grading may be more reproducible in minor gland MEC, though evidence is mixed and limited by small sample sizes [2]. In the present case, the use of both AFIP and Brandwein systems, as recommended by the Royal College of Pathologists and aligned with BAHNO practice standards, provided a more balanced assessment [10]. The discordance between low-grade AFIP and intermediate-grade Brandwein grading is typical and reinforces the need for MDT interpretation, especially as higher-grade disease may prompt more extensive surgery and consideration of neck dissection, increasing morbidity.

Molecular markers are increasingly recognised as useful adjuncts in resolving grading uncertainty. Translocation t(11;19)(q21;p13), resulting in CRTC1/3-MAML2 fusion, is present in approximately 60-80% of MEC and is strongly associated with low-grade histology, indolent behaviour and improved prognosis [11,12]. Cipriani et al. demonstrated that MAML2-positive tumours are more likely to be low grade and have superior outcomes, even when adverse histological features are present [11]. Nakano et al. further confirmed the association between MAML2 fusion status and favourable clinicopathological features across histological variants [12].

Although fusion testing was not undertaken in this case, MAML2 analysis may provide an additional prognostic and diagnostic adjunct in selected diagnostically or histologically challenging MECs. A positive fusion result may support the diagnosis of MEC and provide additional prognostic information when interpreted alongside tumour grade and other adverse pathological features; however, it should not be considered a standalone determinant of treatment. In this case, molecular testing was not considered necessary because the diagnosis was established histologically and management was determined by the combined histopathological, radiological and intra-operative findings. Consequently, MAML2 status would not have been expected to alter the decision to preserve bone.

The second major controversy in palatal MEC is whether the underlying palatal bone should be included as a deep margin. Arguments for bone resection include oncological certainty and avoidance of occult bone invasion, whereas arguments for bone preservation include reduced risk of oronasal fistula, better speech and swallowing outcomes, faster recovery, and avoidance of complex reconstruction, which may otherwise necessitate local flaps or free tissue transfer [8,13].

Radiological assessment of palatal bone involvement in MEC can be challenging. Cortical thinning may represent pressure erosion and reactive remodelling from a slow-growing lesion rather than true tumour infiltration, whereas aggressive invasion is typically associated with cortical breach, marrow signal change, and soft tissue extension into adjacent spaces [9,14]. In the absence of these features, isolated thinning is not generally considered definitive evidence of osseous invasion. This distinction is clinically important, as reactive remodelling does not carry the same prognostic implications as true bone infiltration [13]. In contemporary practice, surgical decision-making increasingly integrates radiological findings with tumour grade, intraoperative assessment of the periosteum, and MDT consensus, and isolated cortical thinning alone does not necessarily mandate bone resection [5,8].

Ord et al. reported no increase in local recurrence among patients with low-grade palatal MEC treated with mucosal excision alone when pre-operative imaging excluded bone invasion, with follow-up extending beyond five years [5]. This supports the possibility that radiologically uninvolved bone can be preserved in selected low-grade tumours. Similarly, Mimica et al. found that prognosis was predominantly associated with tumour grade and stage rather than primary tumour site, with favourable outcomes reported for hard-palate lesions following surgical treatment [6]. Terauchi et al. also reported favourable outcomes in low- and intermediate-grade minor salivary gland MEC, with tumour grade and margin status emerging as important prognostic factors [7]. Although these studies are retrospective and heterogeneous, their findings collectively suggest that recurrence risk is more closely related to tumour biology and adequacy of excision than to routine removal of adjacent uninvolved bone.

In the present case, MDT-guided decision-making favoured bone preservation based on predominantly low-risk histological features, absence of radiological bone invasion, intra-operative confirmation of intact periosteum and bone, and the importance of minimising functional morbidity. The patient’s favourable postoperative course and ongoing disease-free surveillance support current literature suggesting that bone-sparing surgery is an appropriate and safe option in carefully selected cases of low-grade palatal MEC [5-8].

## Conclusions

This case illustrates the challenges of applying different MEC grading systems and integrating histopathological, radiological and intra-operative findings when planning surgery for palatal MEC. Despite discordant AFIP and Brandwein grading, the combination of low-risk pathological features, absence of definitive radiological or intra-operative bone invasion, complete excision and MDT assessment supported preservation of the palatal bone. The patient achieved good short-term functional recovery without postoperative complications or evidence of recurrence during four months of follow-up. While long-term oncological safety cannot be established from a single case, this case supports consideration of bone-sparing surgery in carefully selected low-risk palatal MEC when guided by tumour biology, imaging, intra-operative assessment and multidisciplinary review.
